# Effect of ultraviolet C emitted from KrCl excimer lamp with or without bandpass filter to mouse epidermis

**DOI:** 10.1371/journal.pone.0267957

**Published:** 2022-05-03

**Authors:** Kouji Narita, Krisana Asano, Kyosuke Yamane, Hiroyuki Ohashi, Tatsushi Igarashi, Akio Nakane

**Affiliations:** 1 Department of Microbiology and Immunology, Hirosaki University Graduate School of Medicine, Hirosaki, Aomori, Japan; 2 Institute for Animal Experimentation, Hirosaki University Graduate School of Medicine, Hirosaki, Aomori, Japan; 3 Department of Biopolymer and Health Science, Hirosaki University Graduate School of Medicine, Hirosaki, Aomori, Japan; 4 Ushio Inc., Chiyoda-ku, Tokyo, Japan; Massachusetts General Hospital, UNITED STATES

## Abstract

It has been reported that 222-nm ultraviolet C (UVC) exerts a germicidal effect on bacteria and viruses as well as UV radiation emitted from a conventional germicidal lamp but is less toxic to the mammalian cells than that from a germicidal lamp. An excimer lamp filled with krypton chloride (KrCl) gas principally emits 222-nm UVC. However, the lamp also emits a wide band of wavelengths other than 222 nm, especially UVC at a longer wavelength than 222 nm and ultraviolet B, which cause DNA damage. There are some reports on the critical role of bandpass filters in reducing the harmful effect of UVC emitted from a KrCl excimer lamp in a human skin model and human subjects. However, the effectiveness of a bandpass filter has not been demonstrated in animal experiments. In the present study, mice were irradiated with UVC emitted from a KrCl excimer lamp with or without a bandpass filter. UVC emitted from an unfiltered KrCl lamp at doses of 50, 150 and 300 mJ/cm^2^ induced cyclobutyl pyrimidine dimer (CPD)-positive cells, whereas UVC emitted from a filtered lamp did not significantly increase CPD-positive cells in the epidermis. The present study suggested that the bandpass filter serves a critical role in reducing the harmful effect of emission outside of 222 nm to mouse keratinocytes.

## Introduction

The coronavirus disease-19 pandemic caused by severe acute respiratory syndrome coronavirus 2 (SARS-CoV-2) has made people attend to means and tools to inactivate airborne viruses [1, 2]. Ultraviolet C (UVC) irradiation is getting a lot of attention as a means to disinfect pathogens in public spaces [3–5]. UVC within the range of 240–280 nm is known to elicit a strong germicidal effect, and a low-pressure mercury (Hg) lamp emitting primarily 254-nm UVC has been widely used to kill and inactivate bacteria and viruses [6, 7].

It is well known that 254-nm UVC is directly absorbed by DNA in microorganisms and leads to the formation of dimeric photoproducts between adjacent pyrimidine bases, such as cyclobutyl pyrimidine dimer (CPD). CPD interrupts transcription, translation and replication of DNA, induces mutagenicity and cytotoxicity, and causes bacterial cell death and viral inactivation [8, 9]. However, UV radiation emitted from a low-pressure Hg lamp is also known to be harmful to mammalian cells, to be a human health hazard causing sunburn and dermatitis, and to increase the risk of skin cancer [7, 10].

It has been reported that 222-nm UVC is less toxic to mammalian cells and has a less damaging effect on epidermal cells than UV radiation from a germicidal lamp, because UVC at this wavelength is well absorbed by proteins and/or other biomolecules and reaches only the outmost stratum corneum layer of the epidermis [11]. It has been demonstrated that irradiation with 222-nm UVC at 500 mJ/cm^2^ elicits a bactericidal effect but induces only a small amount of CPD in human skin [12].

UVC is produced by a high-frequency discharge excimer lamp filled with noble gas. Based on the types of noble gases used, excimer lamps emit UVC at specific wavelengths. An excimer lamp filled with krypton chloride (KrCl) gas principally emits 222-nm UVC. However, the lamp also emits low irradiance of 230~280-nm UVC, slight irradiance of ultraviolet B (UVB) and ultraviolet A (UVA) due to electron transitions other than the transition from the KrCl exciplex [13–15]. It is known that these unnecessary emissions and the harmful wavelength components emitted by KrCl excimer lamps are reduced by the use of a bandpass filter. Buonanno *et al*. demonstrated that CPD in a three-dimensional (3D) human skin tissue model is induced by irradiation with UVC emitted from a KrCl excimer lamp without a bandpass filter at a dose of 23 mJ/cm^2^; however, UVC light emitted from the lamp with a bandpass filter is required at a dose of 500 mJ/cm^2^ to induce a small number of CPD-positive cells in the skin models [13].

Some reports have demonstrated that the bandpass filter reduces the harmful effect of UVC emitted from KrCl excimer lamps by reducing CPD formation in the epidermis of human subjects and a human skin model [11, 13, 16]. However, the effectiveness of a bandpass filter attached to a KrCl lamp has not been verified in animal experiments. The present study investigated the effect of UV radiation emitted from a KrCl excimer lamp with or without a bandpass filter on the epidermis of mice.

## Materials and methods

### Mice and ethics statement

Hos:HR-1 (Japan SLC, Inc., Hamamatsu, Japan) is a mutant mouse strain, which is homozygous for the spontaneous *Hr*^*hr*^ mutation. This strain exhibits a hair loss phenotype; however, the mutation does not influence the nature of the skin [17]. In the present study, 7-week-old female Hos:HR-1 mice were used. Mice were maintained under specific pathogen-free conditions at the Institute for Animal Experimentation, Hirosaki University Graduate School of Medicine (Hirosaki, Japan). Animal experiments were carried out in strict accordance with the Guidelines for Animal Experimentation of Hirosaki University. The experimental protocol was approved by the committee on the ethics of the Institute for Animal Experimentation, Hirosaki University Graduate School of Medicine.

### UVC light source

**Fig 1:**
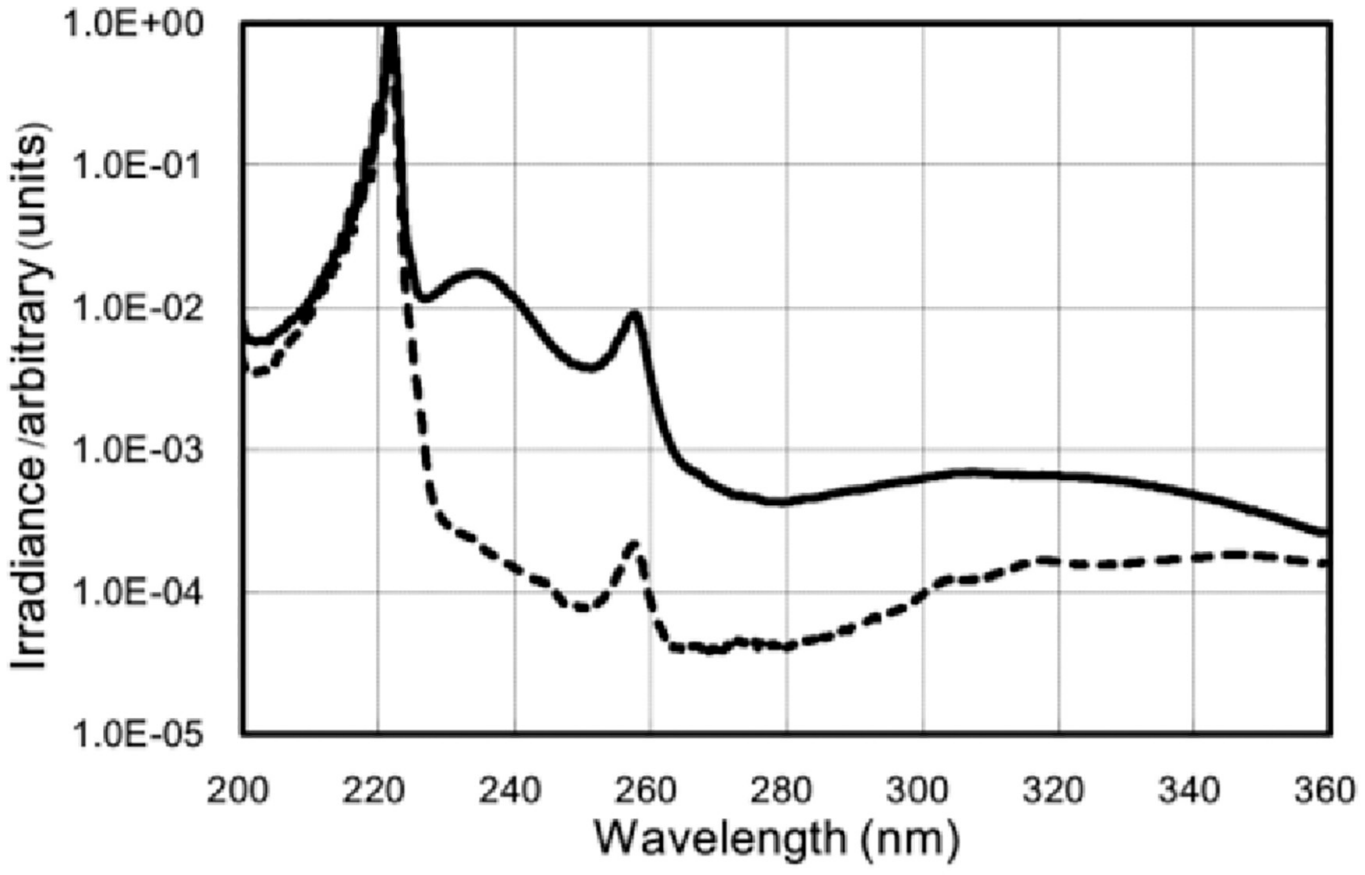
Measured spectra emitted from the KrCl excimer lamp equipped with a bandpass filter or without a bandpass filter. Solid line indicates the semi-logarithmic spectral irradiance of a KrCl lamp without a band pass filter normalized to the 222 nm peak. Dashed line indicates the semi-logarithmic normalized spectral irradiance of the KrCl lamp with a band pass filter that reduces the intensity outside of the 222 nm peak. Devices to measure spectra were described in Materials and Methods section. KrCl: krypton chloride.

Two types of lamp devices were used for UVC light irradiation: One was mounted using a KrCl excimer lamp with an optical filter that restricted spectra emitting light ranging between 200 and 230 nm. The maximum output wavelength of the device was 222 nm. The same type of lamp without that optical filter has a main peak of 222 nm, a broad spectrum derived from KrCl with a peak at 235 nm, a spectrum derived from Cl with a peak at 258 nm, and a small broadband derived from Kr_2_Cl with a peak at 325 nm. The 222 nm-emitting SafeZoneUVC device (Ushio Inc., Tokyo, Japan) is composed of a lamp, air-cooling fan, mirrors and a custom bandpass filter. This multilayer bandpass filter cuts UV radiation from 230 nm to 360 nm so that the spectrum in the UVC and UVB regions of 230 nm and above could be reduced to approximately 1/10,000 of 222 nm as shown in Fig 1. Irradiance emitted by 222-nm light was measured using an S-172/UIT250 accumulated UV meter (Ushio Inc., Tokyo, Japan). The value was found to be 1.40~1.48 mW/cm^2^ at a distance of 100 mm from the emission window and that for the model without a filter was measured to be 1.60~1.68 mW/cm^2^ at the same distance. To acquire positive control data, a conventional low-pressure Hg lamp named SUV-4 (AS ONE Corporation, Osaka, Japan) was used. This lamp emits principally 254-nm UVC and at least 3~4% of UVB that induces more mutagenic CPD than UVC [18, 19]. Irradiance of 254-nm UVC was determined using an S-254/UIT250 (Ushio Inc., Tokyo, Japan). Irradiance of 254-nm UVC was revealed to be 1.43 mW/cm^2^ at a distance of 20 mm from the window. Spectra emitted from the KrCl excimer lamp with the bandpass filter were measured using a multichannel spectrometer QEP01172 (Ocean Insight, Orlando, FL, USA). The QEP 01172 was calibrated with the L7820 D2 lamp (Hamamatsu Photonics K.K., Hamamatsu, Japan) calibrated by the National Institute of Advanced Industrial Science and Technology.

### UVC irradiation to mice

Mice were divided into 10 groups. Each group consisted of 2 mice. Mice were anesthetized by intraperitoneal injection of a cocktail containing medetomidine chloride (Meiji Seika Kaisha, Ltd., Tokyo, Japan), Midazolam (SANDOZ, Tokyo, Japan) and Butorphanol tartrate (Meiji Seika Kaisha, Ltd., Tokyo, Japan) at a dose of 0.3 mg/kg, 4 mg/kg and 5 mg/kg, respectively. Skins of the dorsal surface of hairless mice were irradiated with UVC emitted from the KrCl excimer lamp without a bandpass filter at doses of 10, 15, 20, 50, 150 and 300 mJ/cm^2^ or the KrCl excimer lamp with a bandpass filter at doses of 150 and 300 mJ/cm^2^. UVC irradiation was performed single mouse per exposure. As a positive control, a group of mice were irradiated with UV radiation from a low-pressure Hg lamp, and irradiance of 254 nm in the UV radiation was 30 mJ/cm^2^. Non-irradiated mice were used as a negative control.

### Immunohistochemical analysis

Skin tissues were taken from the dorsal skin of mice belonging to each group immediately after UVC irradiation or non-irradiation and mice were sacrificed by cervical dislocation. The skin tissues were fixed with 10% phosphate-buffered formalin overnight. Paraffin sections (2-μm-thick) were prepared by cutting paraffin-embedded blocks of the skin tissue. After deparaffinization and rehydration of the tissue sections, antigen retrieval was performed by incubating the sections in proteinase K solution (1:1,000; Qiagen GmbH, Hilden, Germany), and the sections were incubated in phosphate-buffered saline (PBS) containing 5% bovine serum albumin (MilliporeSigma, Burlington, MA, USA) for 1 h at room temperature. The sections were then incubated with horseradish peroxidase-conjugated anti-CPD monoclonal antibody (Kamiya Biomedical Co., Seattle, WA, USA) overnight at 4°C. The sections were rinsed three times with PBS for 5 min each at room temperature. The color reaction was developed by the addition of diaminobenzidine and then counterstaining was performed with hematoxylin. The CPD-positive cells and negative cells were quantified by counting the cells in four random high-power (x400) fields for each preparation.

### Statistical analysis

Data are presented as the mean ± standard deviation. The non-irradiated group and each irradiated group were statistically compared using Student’s t-test. P<0.05 was considered to indicate a statistically significant difference.

## Results

### Spectra emitted from the KrCl excimer lamp equipped with or without a bandpass filter

The unfiltered KrCl spectrum has a percent-order spectrum peak in the UVC region of 230~280 nm compared with the 222 nm peak. With the filter, the peak is reduced by about two orders of magnitude. Furthermore, the output power of 235-320-nm wavelengths UVC, UVB without filter is 15.5% of the output power of 200~230 nm wavelengths UVC and the output power of 280~320 nm wavelengths UVC, UVB with filter is 0.43% of the output power of 200~230 nm wavelengths UV (Fig 1).

### UVC emitted from a KrCl excimer lamp without a bandpass filter induces CPD in keratinocytes even at a low dose

**Fig 2:**
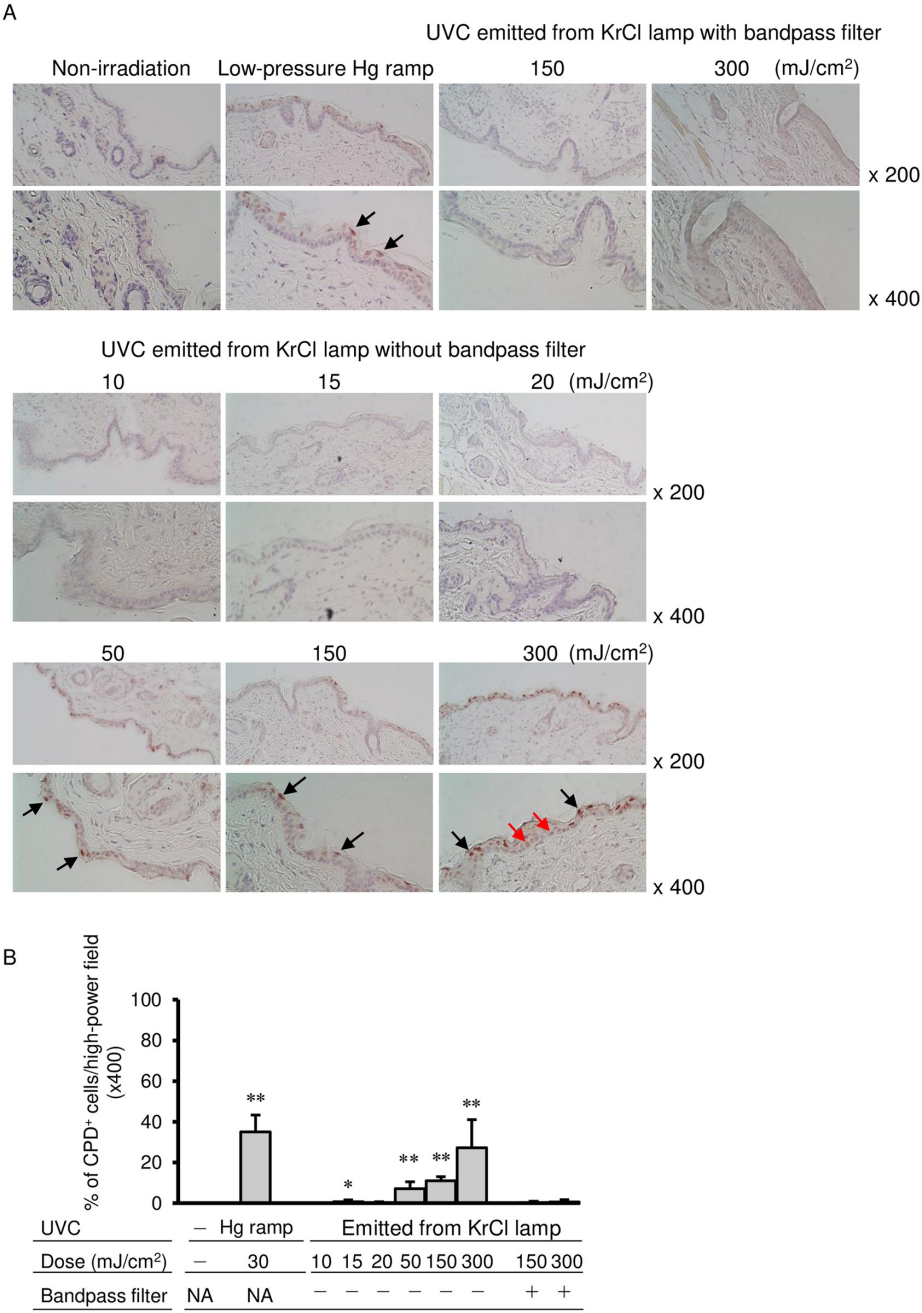
Histological analysis of dorsal skin of mice irradiated with UVC emitted from a KrCl lamp with or without a bandpass filter. The dorsal skin of mice was irradiated with UV radiation from a low-pressure Hg lamp, UVC emitted from a KrCl lamp with a bandpass filter at 150 and 300 mJ/cm^2^ or UVC emitted from a KrCl lamp without a bandpass filter at 10, 15, 20, 50, 150 and 300 mJ/cm^2^. The skin specimens were stained with anti-CPD antibody as described in the Materials and Methods section. Black arrows indicate CPD-positive cells, and red arrows indicate CPD-positive cells in the basal layer of the epidermis (A). CPD-positive and CPD-negative cells were quantified by counting cells in dermis in four random high-power fields (x400) for each preparation, and percent of CPD-positive cells was determined. Data are presented as the mean ± standard deviation. The non-irradiated group and each irradiated group were statistically compared. ** *P* < 0.01, * *P* < 0.05 (B). CPD: cyclobutyl pyrimidine dimers; Hg: mercury; KrCl: krypton chloride; NA: not applicable; UVC: ultraviolet C.

CPD-positive cells were not detected in skin tissues of non-irradiated mice, whereas in dorsal skins of mice irradiated by a low-pressure Hg lamp, ~35% of epidermal cells were CPD-positive cells (Fig 2A and 2B). UVC emitted by a KrCl excimer lamp without a bandpass filter at doses of 10, 15, 20, 50, 150 and 300 mJ/cm^2^ induced 0, 0.75, 0.25, 7.1, 11.0 and 27.3%, respectively, of CPD-positive cells (black arrow) in epidermal cells of mice. CPD-positive cells were also detected in the basal layer of the epidermis irradiated with UV radiation from an unfiltered KrCl lamp at a dose of 300 mJ/cm^2^ (red arrow). UVC emitted by a KrCl excimer lamp with a bandpass filter at doses of 150 and 300 mJ/cm^2^ induced 0.25 and 0.63% of CPD-positive cells in epidermal cells of mice. These percentages were not significantly different from those of non-irradiated mice (Fig 2A and 2B).

## Discussion

Irradiation with 222-nm UVC has recently gained attention as a disinfection method. Furthermore, 222-nm UVC exhibits antimicrobial activity against a variety of pathogens, including SARS-CoV-2, and is as effective as UV radiation from a low-pressure Hg lamp used as a germicidal lamp [3–5]. A low-pressure Hg lamp emits principally 254-nm UVC and UVB at 297, 302 and 313.2 nm. Although the UVB component is <7% of the total UV content, mutagenicity of CPD induced by UVB radiation is greater than that induced by UVC in the epidermis [18, 19]. Unlike UV radiation from a germicidal lamp, 222-nm UVC emitted from a filtered KrCl lamp has been demonstrated to be safe for the skin in animal experiments and human subjects [11, 15, 20–23].

In the present study, irradiation with UV radiation from a low-pressure Hg lamp induced a large number of CPD-positive keratinocytes in the skin of mice. By contrast, UVC emitted from a lamp with a bandpass filter did not significantly induce CPD-positive cells (Fig 2). These results were consistent with previous reports, which demonstrated that irradiation with 222-nm UVC emitted from an excimer lamp with a bandpass filter at a dose of 150 and 157 mJ/cm^2^ did not result in a significant increase in CPD-positive cells in mouse skin compared with that of non-irradiated mice [21, 24]. However, Hickerson *et al*. demonstrated that irradiation with filtered 222-nm UVC at a high dose of 6,100 mJ/cm^2^ induced minimal CPD formation only in the upper layer of the epidermis of human skin samples [15]. Less CPD induction by irradiation with UVC from a filtered lamp has been demonstrated in both animal experiments and human subjects. On the other hand, the effect of UV radiation emitted from the lamp without a bandpass filter on CPD formation has been reported in a human skin model and human subjects. Using a 3D human skin model, Buonanno *et al*. reported that radiant exposures as low as 23 mJ/cm^2^ from the UVC from unfiltered lamp radiation markedly increased CPD formation [13]. Woods *et al*. demonstrated that UVC from unfiltered lamp UVC exposure induced CPD formation in not only suprabasal keratinocytes but also germinal keratinocytes in the basal layer in human subjects [16]. However, the effect of UVC from an unfiltered lamp on CPD formation has not been reported in animal experiments.

In the present study, UV radiation emitted from the lamp without a bandpass filter induced a significant number of CPD-positive keratinocytes at 50, 150 and 300 mJ/cm^2^ in the dorsal skin of mice (Fig 2A and 2B). Mouse epidermis generally comprises only three cell layers and the thickness is <25 μm, whereas human epidermis commonly constitutes 6–10 cell layers with a thickness of more than 50 μm [25]. Although the structure of the epidermis differs between humans and mice, unfiltered 222-nm UVC induced CPD-positive cells in the basal layer of the epidermis of mice as shown in the epidermis of humans (Fig 2A).

It is known that UVC emitted from a KrCl excimer lamp contains 83% of 200~230-nm UVC with the peak emission wavelength at 222 nm. In addition to this range of wavelengths, the lamp spectrum contains 10% of longer-wavelength 230~280-nm UVC, 3% of UVB and 4% of UVA [15]. It has been reported that UVC radiation emitted by an unfiltered KrCl lamp penetrates to the stratum corneum and causes DNA damage in keratinocytes in human skin and a 3D human skin model [13, 16]. In the present study, the bandpass filter reduced 230-280-nm UVC and UVB as shown in Fig 1, while the UV radiation emitted by the KrCl lamp without a bandpass filter, which includes UVC and UVB, elicited a harmful effect in mice.

Increasing evidence has suggested the effectiveness of irradiation with 222-nm UVC for inactivation of airborne viruses and inhibition of airborne and surface-mediated transmission in public locations. However, further studies including animal experiments would be necessary for appropriate use of 222-nm UVC radiation. The present results of mouse experiments suggested that the bandpass filter with a KrCl excimer lamp serves a critical role in reducing the effect of harmful wavelengths on keratinocytes and in increasing the safety of irradiation with 222-nm UVC.
